# ASCT: a pipeline for standardized analysis of MEG/EEG directional connectivity. Practical guidelines for applications of source-based Directed Transfer Function

**DOI:** 10.3389/fninf.2026.1791461

**Published:** 2026-06-23

**Authors:** Miroslaw Wyczesany, Sara Spadone, Tomasz Górski, Adam Łabaza, Michal Domagala, Maciej Kaminski, Tomasz S. Ligeza, Paolo Capotosto, Thomas Kroker, Markus Junghöfer, Stefania Della Penna

**Affiliations:** 1 Institute of Psychology, Jagiellonian University, Kraków, Poland; 2 Departmental Faculty of Medicine, Saint Camillus International University of Health and Medical Sciences (UniCamillus), Rome, Italy; 3 The Faculty of Physics, Astronomy and Informatics, Nicolaus Copernicus University, Toruń, Poland; 4 Center for Cognitive Science, Jagiellonian University, Kraków, Poland; 5 Department of Physics, University of Warsaw, Warsaw, Poland; 6 Department of Neuroscience, Imaging and Clinical Sciences, University “G. d’Annunzio”, Chieti, Italy; 7 Institute for Advanced Biomedical Technologies (ITAB), University “G. d’Annunzio”, Chieti, Italy; 8 UdA-TechLab, Research Center, University “G. d’Annunzio”, Chieti, Italy; 9 Institute for Biomagnetism and Biosignal Analysis, University of Münster, Münster, Germany

**Keywords:** directed connectivity, source reconstruction, leakage correction, EEG, manual, recommendations, standardization, replicability

## Abstract

The article presents the Atlantis Source Connectivity Toolbox (ASCT), a pipeline for the complete analysis of source-based directed (effective) connectivity from MEG/EEG signals. Connectivity estimation is implemented with the Directed Transfer Function, a multivariate autoregressive method grounded in the Granger causality approach. The theoretical concepts and challenges in source reconstruction and causal inferences from electrophysiological and biomagnetic recordings are introduced. Importantly, the guidelines for the custom analyses are also presented together with the toolbox user manual. By providing the complete analytic pipeline with practical recommendations, we intend to support standardization and replicability in connectivity studies. The validation of the method is further demonstrated with simulated MEG data, prepared from real resting data recording, as well as with realistic EEG data from the Eriksen flanker task. Our results highlight the importance of routine use of the leakage correction that prevents the appearance of spurious links. We also discuss potential benefits of source separation prior to their reconstruction for more accurate estimates of connectivity.

## Highlights

- A pipeline for source-based analysis of directed connectivity is proposed
- Guidelines for setting up custom analyses are provided
- Tests show the importance of leakage correction and the benefits of source separation.

## 1 Introduction

Network analysis, which can reveal a dynamic information exchange between cortical areas, is a key to understanding how the brain guides our behavior. In recent years, connectivity investigations have been dominated by the fMRI technique, which allows us to see the brain in action as a system of collaborating nodes characterized by a constant exchange of information. Indisputably a milestone for neuroscience, this approach comes with some significant limitations. Firstly, the poor temporal resolution of the BOLD response prevents it from studying fast and dynamic changes in neural communication, which is crucial for analyzing short-lived and transient cognitive processes. Furthermore, fMRI struggles to quantify the asymmetry of reciprocal interactions which comprises the fundamental aspect of the brain connectome. This directed (or effective) connectivity (DC) helps us identify top-down or bottom-up mechanisms linking primary and high-order regions during specific tasks or at rest (Geweke, 1982; Schreiber, 2000; Rosas et al., 2019). Importantly, these constraints can be overcome by analyzing EEG and MEG signals, which directly reflect the activity of cortical cell populations and are better suited to capture the directional and dynamic nature of neural communication.

Numerous approaches to EEG/MEG connectivity have been proposed so far. One of the most popular concepts for measuring directionality in the time domain is Granger Causality (Granger, 1980), widely applied to fMRI (Bressler et al., 2008; Spadone et al., 2015) and MEG/EEG data (Marinazzo et al., 2011). In turn, in the frequency domain, the Generalized Partial Directed Coherence (GPDC) (Baccala et al., 2007), and the Directed Transfer Function (DTF) (Kamiński and Blinowska, 1991) have been developed, both based on the Fourier transform of the MVAR model coefficients. DC methods typically require advanced processing of the signal with many computational steps. Each of them compels decisions on numerous parameters that significantly affect the final outcomes. Improper settings or even default values can often result in inaccurate output. Unfortunately, the impact of these decisions is usually not sufficiently documented or even determined, which poses a real challenge to the reliability and reproducibility of DC estimation. Indeed, a review of the literature reveals vast variability and randomness in the ways the connectivity is estimated, which strongly deviates from the desired methodological rigor (Bastos and Schoffelen, 2016; Chiarion et al., 2023; Wyczesany et al., 2025b). Therefore, the primary aim of the current paper is the standardization of DTF-based DC analysis by providing practical guidelines for data processing. The guidelines are supported by the results of simulations and real data analyses reported in this work. Certainly, the striving for scientific replicability emphasizes the need for well-controlled analytic methods to yield reliable conclusions. Fortunately, new initiatives are being undertaken to provide guidelines and limit the decisive degrees of freedom when analyzing electrophysiological and biomagnetic data (Jas et al., 2018). Subsequently, the first part of the IFCN recommendations has been released (Babiloni et al., 2020), covering some classic analytic methods for the resting-state data. Importantly, the authors notice that the standardization problem is particularly pronounced for the connectivity methods due to their sophisticated methodology and complex, multi-stage signal processing. So our secondary aim is to familiarize readers with these concepts necessary for full data processing.

Following these concerns, we propose a complete pipeline for effective connectivity analysis of EEG/MEG data, implemented as the Atlantis Source Connectivity Toolbox (ASCT). The toolbox covers the whole process of signal processing, from raw data to DTF estimation, statistics, and visualization of results. After setting up the analysis, i.e., defining the experimental design and adjusting parameters, a fully automatic mode of operation is available. The process can still be supervised at all stages with a data viewer and log files. Importantly, to enable reliable analysis of users’ own data, we provide recommendations regarding the parameter settings and describe their impact on the final results (section “4 User manual and guidelines” of the paper). Our method underwent extensive testing not only with real experimental data but also using simulated and realistic signals, with some examples presented below that can support the usability of our approach.

## 2 Methods

### 2.1 Overview

The Atlantis Source Connectivity Toolbox (ASCT) is an open-source software designed for a complete analysis of directional connectivity on EEG and MEG datasets. It has been released under the GNU General Public License and can be downloaded from https://atlantis.psychologia.uj.edu.pl. The ASCT works in the Matlab environment, and it partly relies on other open-source toolboxes, with FieldTrip being the most important (Oostenveld et al., 2011). Other necessary external functions are shipped within the ASCT for user convenience and include the code of EEGlab (Delorme and Makeig, 2004), fastICA (Hyvärinen and Oja, 2000), SimBio (Vorwerk et al., 2018), HCP Workbench (Larson-Prior et al., 2013), AAR (Gómez-Herrero, 2007), ROInetworks (Colclough et al., 2015), and Multar (Kaminski et al., 2005).

**FIGURE 1:**
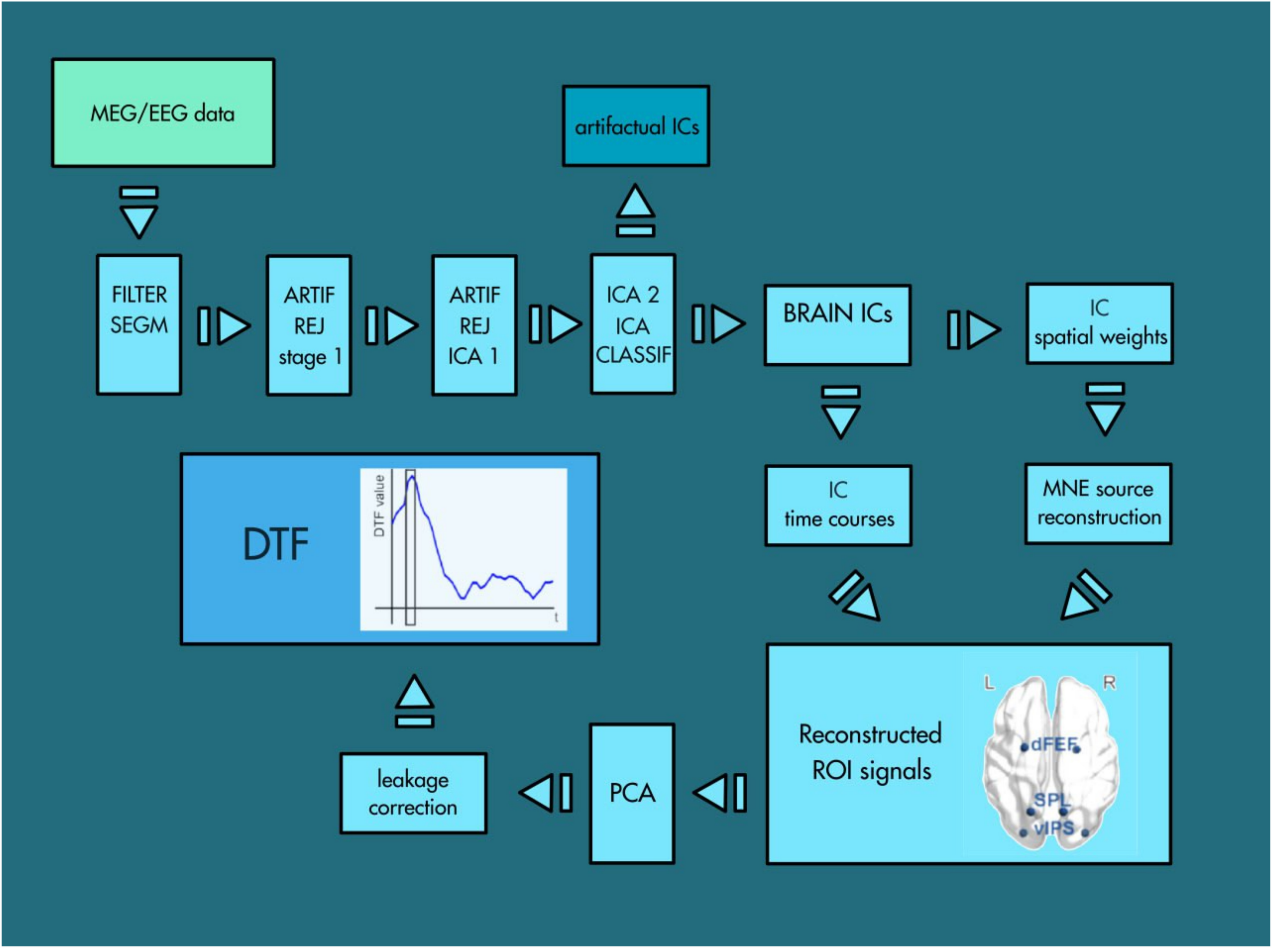
The analytic pipeline implemented in the ASCT.

Data processing is partially based on the established pipeline described in Mantini et al. (2011) and extensively applied to the analysis of MEG activity (de Pasquale et al., 2012; Larson-Prior et al., 2013; de Pasquale et al., 2021; Spadone et al., 2021a). Preprocessing steps include filtering, data segmentation, and multistage artifact rejection (AR). This latter step begins with the removal of bad channels and trials based on the sensor-level detection of significant deviation from typical signal parameters. Subsequently, Independent Component Analysis (ICA) is used to decompose the remaining data into possibly independent signal sources (known as independent components; ICs). Firstly, the decomposed signal is used for further detection of artifactual trials that are still visible at the level of IC time courses. Then, remaining noisy sensors can be detected based on strong dissimilarities with neighboring channels, using IC topographic maps. After that, cleaned data are subject to another (“clean”) ICA decomposition. Resulting ICs are classified into those of the brain and of non-brain origin (considered artifacts) with pre-trained machine learning models. Only brain components are retained and localized using their topographies with the depth-weighted minimum norm estimate method (wMNE). Individual head and source models based on T1 MRI scans and realistic sensor placement are preferably applied here, if available. Brain activations in the selected regions of interest (ROIs) are reconstructed as the superposition of all brain IC time courses. The resulting three-dimensional data that include three spatial components are then flattened to represent the course of focal activation by retaining the first principal component. Then, the multivariate leakage correction is applied to the reconstructed ROI signals to minimize the impact of spatial blur on reconstructed sources. Eventually, the effective connectivity is estimated using the DTF method. The final stage includes statistical testing and visualization of connectivity maps. The complete analytic pipeline implemented in the ASCT is shown in Figure 1

### 2.2 Preprocessing

The initial preprocessing includes the import of MEG/EEG data and its filtering using windowed-sinc FIR filters (Widmann, 2012). Prior to artifact detection and rejection, the signal is segmented according to the events defined by the experimental design. This allows for estimating the unbiased statistical parameters of channels and trials that do not include signal portions other than those related to experimental tasks.

Much attention was paid to the multi-staged process of artifact rejection. Initially, bad sensors are detected and removed based on the distribution of channel variance values. In the case of EEG signals, bad channel detection can benefit from including EOG (oculographic) derivations. This would allow for temporary correction of eye movements to minimize their bias on the statistics of frontal electrodes (He et al., 2004). For EEG only, the signal is now re-referenced to the average of all remaining scalp sensors. Next, bad portions of data are removed in the subsequent steps according to excess trial variance, the difference between min/max trial amplitude, and muscle activity contamination (estimated as elevated power for higher frequencies).

### 2.3 Independent Component Analysis (ICA)

During the ICA procedure, the sensor signals are decomposed into potentially independent sources. In this step, we switch from the sensor signals (measured by electrodes, magnetometers, or gradiometers) to the representation of the original recording by the set of independent components (ICs). Each IC is characterized by its own signal time course and scalp topography. Hence, the signal measured by sensors, *y*, can now be represented by a mixture of ICs (Equation 1):


y=Ms (1)

where *s* represents IC signals in time. That is why the M matrix, which provides the transformation between both representations, is called a mixing matrix. In the basic ICA implementation, the number of resulting components (*n*_*c*_) is equal to the number of input sensors (*n*_*s*_), and the mixing matrix remains square. Typically, the resulting components are ordered according to their decreasing power, so the lower the IC number, the more information it conveys. In practice, the number of relevant ICs that are sufficient to represent the original recording is smaller than *n*_*s*_. In the ASCT, the FastICA algorithm (Hyvärinen and Oja, 2000) is implemented with a deflation scheme enabled. It yields a reduced number of ICs, which, however, can still accurately represent the data.

The rationale for decomposing EEG/MEG recordings with ICA is twofold, as already discussed in Mantini et al. (2011). First, it represents a data-driven approach to efficiently separate and reject artifacts. Secondly, separated components of brain activity provide possibly unitary and focused sources that can be more efficiently localized with available methods. These major points reflect two stages, where ICA decomposition is performed in the ASCT. The first decomposition (ICA1) is applied to detect artifacts that are still present in the signal after sensor-space artifact rejection. This is accomplished by analyzing the IC signal time course for bursts or excess variance to remove artifact-contaminated trials. Additional removal of noisy channels is also carried out by detection of ICs that contribute to a single sensor only. This means a very steep gradient around a single channel on topographical maps. It should be noted that the analysis of ICA1 timecourses and topographies is only used for detecting potentially contaminated trials and channels. Having them marked, the removal is performed on the sensor level. After the preliminary stages of artifact rejection, ICA2 can provide a finer decomposition as it runs on relatively clean signals. The ICA2 step is completed with the classification of the resulting ICs into those of brain and non-brain origin. Only the former ones, representing cortical activity, are retained.

As ICA is a non-deterministic procedure, the results obtained with the same data vary from run to run (due to the initiation of the separation with pseudorandom guesses). In order to obtain a fine separation, ICA2 is repeated multiple times, and the optimal run is selected for further analysis. The choice is based on the metric that maximizes the number of brain ICs and minimizes their artifact contamination. To estimate artifact contamination, additional reference electric channels like EOG and ECG are required. This parameter measures how well artifacts were separated into the non-brain components and is defined as 1−mean(H_brainIC_), where H_brainIC_ is a vector with elements H_i_ = max(corr(p_elec_, p_brainIC_i__)), representing the power time courses of the reference electric channels and the power of i-th brain-classified component, respectively [see: (Mantini et al., 2011; Larson-Prior et al., 2013) and the Human Connectome Project Software].

#### 2.3.1 Classification of independent components

The purpose of this step is to provide unsupervised classification of the resulting components into those that indicate brain activity and non-brain components that represent various sources of noise (environmental, physiological, e.g., cardiac, ocular, and residual channel artifacts). Machine learning has been previously used to classify ICA components, but typically utilizing either simple features of the signal (Sai et al., 2018), or using raw ICA component topographies or signals with convolutional neural networks (Croce et al., 2019; Treacher et al., 2021). Here, we draw from the experience of both of these approaches, using two streams of processing: one designed to extract selected signal parameters and the other utilizing ICA topography maps. Hence, two models account for different signal features, with the joint probability considered finally via weighted average. Both models were trained on manually annotated datasets by experts from privately sourced databases using over 5.5k samples. We utilized a validation set consisting of 30% of our training set for hyperparameter tuning and network configuration testing.

The first model utilizes a simple, custom-built feed-forward network, operating on a repertoire of seven or eight signal metrics. Three of them (correlations between electric channel signals and IC time course, between their power, and between their spectral densities) help to identify ocular artifacts and those stemming from extra-cerebral electrical activity in the body. Kurtosis is effective at detecting transient, high-amplitude, and sparse artifacts like muscle and motion artifacts. The next two features, 1/f spectrum similarity and spectral flatness are meant to track down general noise components. Regular peak detection can account for heart-based components. Additionally, for event-related analysis, post-stimulus signal variability was introduced. The classification network itself consists of two Fully Connected layers, consisting of 50 and 100 units, respectively, each followed by batch normalization and the Rectified Linear Unit (ReLU) activation function. The network ends in a Softmax layer to gather the probability of brain and non-brain origin of a given IC. The number of nodes in fully connected layers, the optimization algorithm, and the regularization parameter were chosen based on grid search cross-validation on the validation dataset for each new instance of a model. The best solver proved to be root mean square propagation with a learning rate of 0.001.

The second model is a custom-built convolutional neural network (CNN) designed to extract spatial (topographical) features of ICs. It consists of four convolutional units (with respective layer sizes of 16, 32, 32, 64 and filter sizes of 3, 5, 5, 5), each composed of a convolutional layer, a ReLU activation function, and a max-pooling layer to reduce output dimensionality. After feature extraction, classification is performed using two fully connected layers with 2000 and 1300 nodes, respectively. Each layer is followed by batch normalization (to improve training stability) and a ReLU activation function. As a final output, we utilize a Softmax layer to estimate class probabilities. We explored in a grid-search fashion, different initial learning rates (0.01, 0.003, 0.001, 0.0003), different optimizers (rmsprop, Adam, sgdm) and different L2 regularization rates (0.001, 0.0005, 0.0001). Finally, the network was trained using the Adam optimizer (Kingma and Ba, 2017) and L2 regularization parameter of 0.001. Additionally we utilized a piecewise learning rate scheduler with an initial learning rate of 0.001.

**FIGURE 2:**
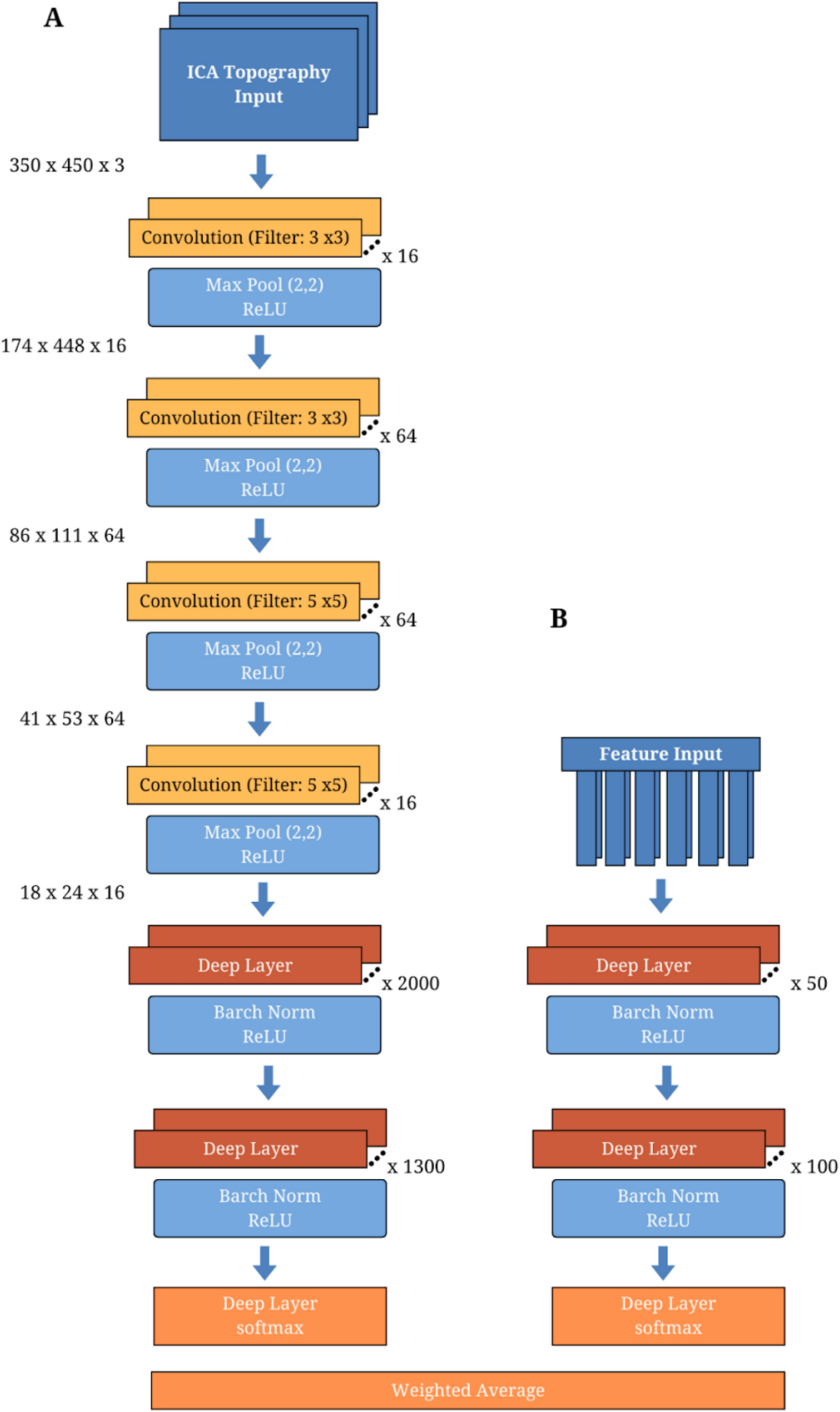
Custom ICA classifier (fitted for EEG, non-resting state data) consisting of: **(A)** Convolution-based model extracting features from ICA topographies. **(B)** A simple classifier based on spectral and statistical ICA features.

Since MEG and EEG topographic components and their characteristics differ, separate models were trained. The classifier used by the ASCT, with all layer parameters is graphically presented in Figure 2.

### 2.4 Source localization

#### 2.4.1 Neural activity and forward calculations

The neuronal postsynaptic potentials and the resulting transmembrane primary currents *j_p_* of a large population of neurons are balanced on a macroscopic scale by volume currents *j_v_* flowing outside the cells. For MEG, where the head is typically modeled by a homogeneous medium, only postsynaptic currents count as the ultimate source of the signals measured over the scalp. For EEG, postsynaptic currents are also the main source of the signal, with some notable contribution of the volume currents as well. The synchrony in the larger population of pyramidal neurons is necessary to produce the currents strong enough to be detected. See the Appendix for more details on the origin of MEG and EEG signals.

In practical implementation, primary current dipoles in the cortex are modeled as a dense net of discrete sources, which is referred to as a *source model*. The number of source dipoles (*n*_*p*_) is typically of the order of 10–30 thousand. To account for cortical folding and to consider that each source dipole represents the activity of a cortical patch or volume, each source dipole is described by the 3D activation vector representing three spatial components of activation (x, y, z). The joint activations of all source dipoles model the activation of the whole neocortex.

**FIGURE 3:**
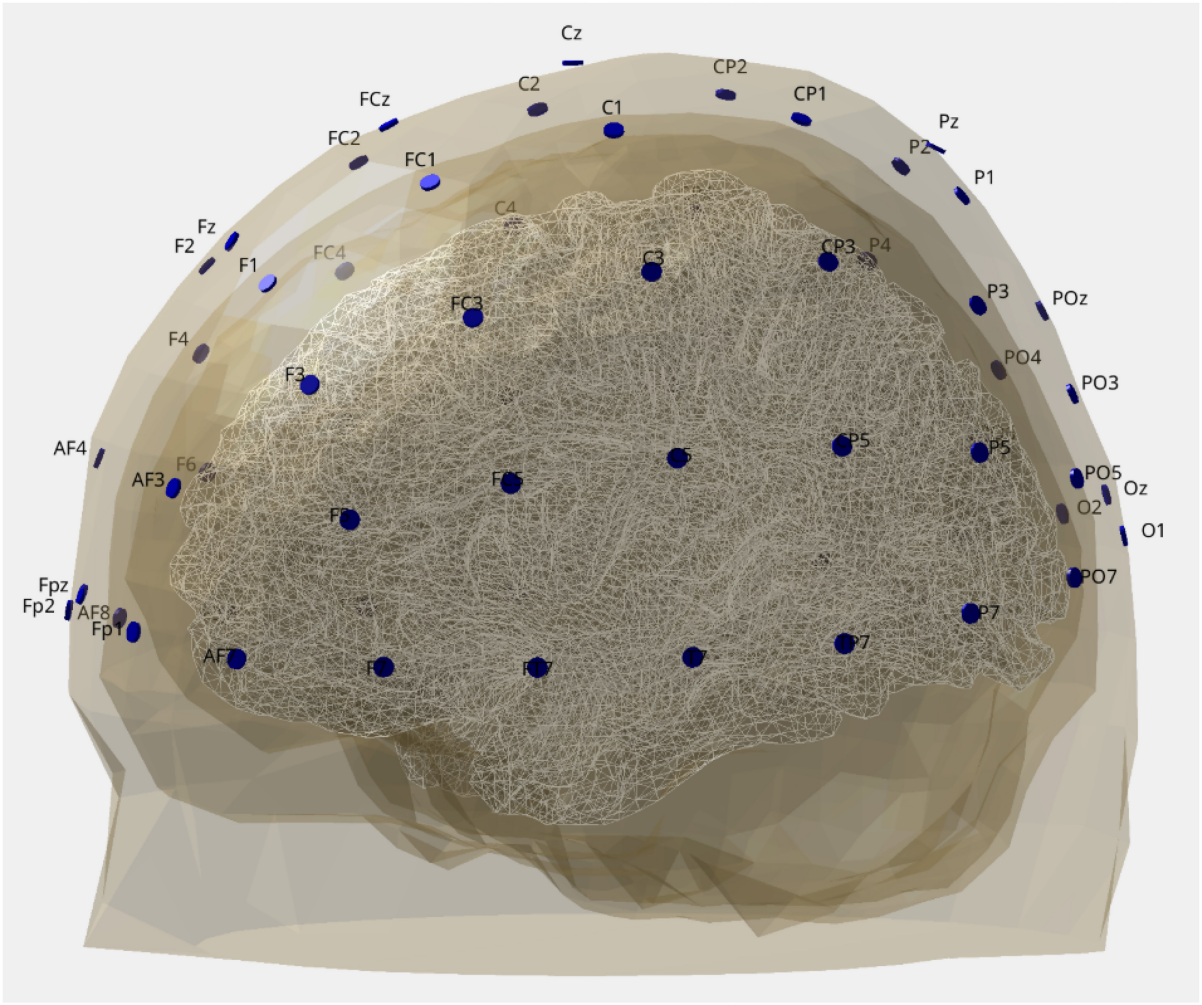
Realistic source and head models created from the SPM8 template brain with electrode placement marked. The five-compartment SimBio head model is represented by skin, skull, cerebrospinal fluid (CSF), gray matter, and white matter, all built from a large number of small pyramids. Inside, the source model represents the cortical issue with source dipoles located on pyramids’ vertices.


In order to reliably determine the propagation of an electric/magnetic field in the head, we need a geometrical representation of head tissues and their electric properties, called a head model. With a given head model and sensor set, this computation, known as a forward problem, yields a leadfield (or gain) matrix *L*. The leadfield of size *n*_*s*_ × *n*_*p*_ × 3 tells how strong the activations of a unitary source dipole are visible at each measurement sensor *y*. Sample individual head and source models are presented in Figure 3.

y=Lx (2)

#### 2.4.2 The inverse problem

The inverse problem, which is an actual challenge for source reconstruction, describes finding the activations of cortical source dipoles that underlie the measured (therefore known) MEG or EEG signal. This is, however, a non-trivial problem due to the high number of unknown source dipoles *x* that strongly outnumbers the known sensor signals *y*. This can result in an infinite number of computationally valid solutions (many possible source activations can provide the same measured field) that need to be constrained. In the ASCT, the minimum norm estimate (MNE) with depth weighting is implemented as a default method (Wang et al., 1992; Hämäläinen and Ilmoniemi, 1994) (using other localization methods is also possible, but we do not recommend them, as no sufficient validation works were performed within the ASCT framework).

Therefore, to determine the x term for Equation 2, while both y and L are known, the following function is minimized:


$$F\,\left(x\right)={\left|\mathrm{Lx}-y\right|}^{2}+\lambda \,{\left|x\right|}^{2}$$ (3)

Here, the first term is an error term, which tells how far our hypothetical source distribution lies from the distribution that generated the measured potentials. The second term contains a regularization parameter λ, which helps to choose among many possible solutions by penalizing the overall energy of the sources and avoiding overfitting. The preference toward low-energy solutions reflects the evolutionary constraints on the organisms’ energy expenditure as an important survival advantage. The regularization stabilizes the solution of the ill-posed inverse problem by imposing a preference for source distributions with minimal Euclidean norm, thereby selecting a unique and numerically stable solution from the otherwise infinite set of valid reconstructions.

The function from Equation 3 is a convex function; therefore, its local minimum is also a global minimum and is given by:


$$\hat{x}=L^{T}\,{\left({\mathrm{LL}}^{T}+\lambda \,I\right)}^{-1}\,y=L_{\lambda }^{+}\,y$$ (4)

where $\hat{x}$ is the estimate of source dipole activations (a hat over a variable will be further used for denoting estimates).

#### 2.4.3 Depth weighting

The classic MNE favors superficial source locations over those located deep in the head. To counteract this bias, a modification scheme called depth-weighting is applied (Lin et al., 2006), which applies to the $L_{\lambda }^{+}$ term. Details are provided in the Appendix.

#### 2.4.4 Localization of independent components

In the ASCT, determining cortical activation is based on prior localization of ICs (Mantini et al., 2011; Larson-Prior et al., 2013). In other words, the sensor maps (topographies) of ICs are the exclusive input to the source localization equations. This method can be beneficial compared to localizing complex brain activity consisting of multiple concomitant sources, especially when the sources significantly differ in their strength of activation (Hauk et al., 2011). Thus, MNE can be more accurate with simplified input that is provided by IC separation. Hence, for each IC, we feed the *y* term in Equation 4 with its normalized topographic weights (between −100 and 100) instead of the raw sensor signal values. Therefore, the inverse solution ${\hat{x}}_{i}^{m}$ is also expressed in terms of weights (Equation 5):


$${\hat{x}}_{i}^{m}=L^{+}\,\left(\lambda_{i}\right)\,m_{i}$$ (5)

where *m_i_* is a mixing matrix column and λ_*i*_ is the individual regularization parameter, both referring to the current IC.

The *λ_*i*_* parameter affects the accuracy of source reconstruction. It is estimated using the SNR of the individual IC (Lin et al., 2006). The SNR is based on the ratio between the individual global field power of the scaled (between −100 and 100) IC sensor map and the calibration noise on the same maps. Its optimal value is linked to the specific MEG system, and the way of its estimation is covered in the section “4 User manual and guidelines.”

### 2.5 Signal reconstruction

After localizing all brain ICs, it is possible to calculate brain activations in each dipole of the source model. The three directions of the MEG vector activity are estimated at each ROI as the linear combination of the non-artifactual IC time courses weighted by the related source map values, according to the following formula:


$${\hat{q}}_{i_{x}}\,\left(t\right)=\sum_{i=1}^{m_{b\,c}}q_{j_{x}},i\propto_{i}{\hat{s}}_{i}\,\left(t\right)$$ (6)

$${\hat{q}}_{i_{y}}\,\left(t\right)=\sum_{i=1}^{m_{b\,c}}q_{j_{y}},i\propto_{i}{\hat{s}}_{i}\,\left(t\right)$$

$${\hat{q}}_{i_{z}}\,\left(t\right)=\sum_{i=1}^{m_{b\,c}}q_{j_{z}},i\propto_{i}{\hat{s}}_{i}\,\left(t\right)$$

where *m*_*bc*_ is the number of brain (non-artifactual) independent components, $\left[{\hat{q}}_{i_{x}}\,{\hat{q}}_{i_{y}}\,{\hat{q}}_{i_{z}}\right]$ is the output of the source reconstruction along the three Cartesian directions, *α_*i*_* is the amplitude restoring factor computed to normalize the IC sensor map in the range [−100, 100] before projection and ${\hat{s}}_{i}$ is the matrix of the brain IC time courses (*m*_*bc*_ × time).

Equation 6 yields signals that contain three spatial components (x, y, z), representing varied orientations of source dipoles due to the cortical folding. Before connectivity is estimated, these three dimensions are subject to PCA (principal component analysis). Only the first temporal component is retained to represent the overall (orientation-independent) measure of activation in time. This is justified by the anatomical properties of the cortex, where adjacent neurons are similarly oriented.

In the ASCT, the choice of the regions of interest (ROIs) is defined by the coordinates in the standard MNI space. As the dipole net is discrete, the required ROIs have to be estimated by the nearest dipoles existing in the source model. Moreover, when realistic source models are used, ROI coordinates are individually warped to fit participants’ brains before choosing the exact source dipole in the net.

#### 2.5.1 Leakage correction

Finally, before calculating the connectivity, a leakage correction is applied. Leakage refers to the fact that the spatial extent of sources localized with MNE tends to grow (“leak”) compared to actual cortical activation. This introduces some crosstalk (zero-lag correlations) between independent sources. Although directed connectivity is often considered leakage-free by definition, in practice, samples at time *t* are somehow linked to samples at previous timepoints. This may affect MVAR model coefficients (see below), which would result in connectivity estimation bias. In the ASCT, a symmetric multivariate orthogonalization procedure is applied (Colclough et al., 2015). This correction is suitable for handling multiple ROIs at the same time, which differs from the pairwise leakage correction approaches, e.g., (Brookes et al., 2012; Wens et al., 2015; Betti et al., 2018), and is suitable for MVAR models.

### 2.6 Directed connectivity estimation

Having the ROIs signals reconstructed, we can now perform an estimation of their interdependence. To estimate the effective connectivity, which describes causal relations in the dataset, we first need to adopt a definition of causality. The Granger causality approach (Granger, 1969) is widely used in biomedical data analysis. It defines a signal *S2* as causal for a signal *S1* if values of *S1* can be better predicted using previous values of both signals *S1* and *S2* rather than using previous values of signal *S1* alone.

The ASCT uses the Directed Transfer Function (DTF) (Kamiński and Blinowska, 1991; Blinowska et al., 2004) as a primary method for evaluating connectivity (although other methods like Partial Directed Coherence are also possible). It is a parametric method based on fitting a multivariate autoregressive model to the data. It assumes that a sample of the data in *k* channels at time *t* can be expressed as a weighted sum of *p* previous samples with a random component added (Equation 7):


$$X\,\left(t\right)=\sum_{j=1}^{p}A\,\left(j\right)\,X\,\left(t-j\right)+E\,\left(t\right)$$ (7)

where *X*(*t*) is the vector of data values at time *t*, and *E*(*t*) is the vector of random component values at time *t*. The matrices *A*(*j*) of size *k* × *k* are known as the model coefficients, and *p* is called the model order. This parameter determines how many samples in the signals’ past are used for prediction purposes. The way of estimating its optimal value is further described in the section “4 User manual and guidelines.” During the fitting of the model parameters, we estimate *A*(*j*) parameters, which combine previous samples from all the signals to minimize the variance of the *E*(*t*) component, which may be interpreted as the prediction error of signals *X*(*t*).

The MVAR model can be transformed to the frequency domain (Equation 8), where it takes the form of a linear filter *H* (Equation 9) with the white noise vector *E* on the input and the signal vector *X* on its output:


$$X\,\left(f\right)=A^{\left(-1\right)}\,\left(f\right)\,E\,\left(f\right)=H\,\left(f\right)\,E\,\left(f\right)$$ (8)

$$H\,\left(f\right)={\left[\sum_{m=0}^{p}A\,\left(m\right)\,e\,x\,p\,\left(-2\,\pi \,i\,m\,f\,\Delta \,t\right)\right]}^{-1}$$ (9)

where *f* is frequency, *X*(*f*), *A*(*f*), and *E*(*f*) are the Fourier transforms of *X*(*t*), *A*(*t*), and *E*(*t*), respectively, and Δ*t* is the data sampling interval. The matrix *H*(*f*) = *A*^–1^(*f*) is known as the transfer matrix.

Based on the transformed model, we can calculate spectral functions as well as functions describing the relationships between data channels. MVAR modeling is a truly multivariate approach, and the obtained spectral estimates are calculated using information from all the signals simultaneously. This way, they are free from the interpretation pitfalls possible in pairwise analysis (Bastos and Schoffelen, 2016; Babiloni et al., 2020). The non-normalized DTF function is defined in the frequency domain as (Equation 10):


$$\gamma_{i\,j}^{2}\,\left(f\right)={\left|H_{i\,j}\,\left(f\right)\right|}^{2}$$ (10)

where H_*ij*_(f) are elements of the transfer matrix H. It describes the causal influence of channel j on channel i at frequency f.

### 2.7 Statistical testing

Group statistics in the ASCT are limited to simple contrasts between predefined configurations of conditions, groups, and sessions. As connectivity distribution typically deviates from normality, a Wilcoxon signed-rank test is used by default. Before statistical testing, it is possible to screen for outlying values in the data using the interquartile (IQR) distribution cut-off.

## 3 Experimental validation

To demonstrate the reliability of the ASCT pipeline, two sample analyses are presented. The first one was a simulation based on realistic MEG data where the dependencies between sources were artificially introduced by “injecting” the previously modified brain signals into the head model. This resulted in a mock MEG recording with known brain sources and controlled connectivity structure. The second analysis is a single-subject EEG recording from the Eriksen flanker task. This task was chosen, as its underlying neural mechanisms are well described and agreed upon.

### 3.1 Simulations on realistic RS MEG data

#### 3.1.1 Data preparation

In order to use the realistic brain-like signals, the data used in the simulation were prepared from the real resting-state single-subject MEG recording during fixation lasting 288 s. Data were recorded with a 1025 Hz sample rate using a whole-head system comprising 153 channels operating at the University of Chieti (Della Penna et al., 2000; Pizzella et al., 2001), being a part of the procedure approved by the Institutional Review Board and Ethics Committee of the University of Chieti (EU project HEALTH-F2-2008-200728; 22.07.2008). Institutional guidelines and privacy rights of the participant have been observed, and informed consent was obtained. After preprocessing and ICA decomposition using custom software for the analysis of resting state (Favaretto et al., 2021; Spadone et al., 2021a), we estimated the activity of selected MEG voxels comprised in the main parcels of the default mode network (DMN) in the following ROI locations: left medial prefrontal cortex (LMPFC; MNI: −13, 52, 23), right angular gyrus (RAG; MNI: 51, −64, 32), left angular gyrus (LAG; MNI: −43, −76, 35), and precuneus/posterior cingulate cortex (PCC; MNI: −3, −54, 31) as in De Pasquale et al. (2010).

Before these realistic ROI signals were used for the simulations, they were cut into four equal parts of length 72 s and shuffled to remove any intrinsic interdependence. The controlled connectivity between regions was then introduced by the selective addition of time-shifted signals from other ROIs with different proportions (Equation 11):


$$S_{i}^{\left(c\right)}=\left(1-C_{i}\right)\,S_{i}+\sum_{j=1}^{N}c_{i\,j}\,S_{j}\,\left(t-\xi_{i\,j}\right)$$ (11)

where $S_{i}^{\left(c\right)}$ are the resulting signals with connectivity introduced; *S_i_* are the original source signals; *c*_*ij*_ are the mixing coefficients, 0 ≤ *c*_*ij*_ ≤ 1, *c*_*ij*_ = 0, *c*_*i*_ = ∑_*j*_*c*_*ij*_; ξ_*ij*_ are the time shifts; and *N* is the number of signals. In the current simulation, we used four signals, where we introduced connectivity with the following dependency structure:

[(1, 2, 50 ms, 0.1), (3, 4, 30 ms, 0.1)],

The above indicates two relationships added: signal 1–2 and signal 3–4 with relative strengths of 0.1 and time lags of 50 ms and 30 ms, respectively, as guided by the realistic cortical propagation rate (Basti et al., 2019). A custom homogeneous individual head model based on the participant’s T1 scan was created. The source model was obtained with the ASCT script using FreeSurfer cortical reconstruction downsampled by the HCP Workbench, which resulted in a net containing 15684 dipoles. The prepared source signals were injected into four selected ROI locations. Simulated brain sources were spherical with a radius of 1 cm, with the full signal in the center of the sphere, decreasing gradually to zero with the rising distance from the centroid according to the bell curve. Finally, forward calculation using the individual head model was performed, which resulted in simulated MEG signals featuring 153 magnetometers.

#### 3.1.2 Data analysis

The simulated MEG recording was filtered with the windowed sinc finite impulse response (FIR) filters: high-pass (freq 2 Hz, order 6766) and low-pass (freq 48 Hz, order 1000) and downsampled to 128 Hz. The artifact rejection stage was skipped, as the original signals were extracted from already refined brain components. The fastICA algorithm with *tanh* non-linearity and iteration number set to 200 was applied to decompose simulated data. Topographies of ICs were subject to source reconstruction based on an individual head and source model using the MNE method with the depth weighting *d* parameter set to 0.5 and the noise level equal to 7, a value previously determined for this system. ROI signals were reconstructed from ICs, and the first component derived from the PCA was retained. Next, the leakage correction was performed. Both uncorrected and corrected ROI signals after virtual segmentation into chunks of 4 s length were subject to connectivity estimation using the non-normalized DTF method with model order equal to 7 in the theta to gamma range. Additionally, the connectivity of source signals that were used in the simulation was also estimated with the same DTF parameters. Both raw and processed files are available at https://tlantis.psychologia.uj.edu.pl/data/Sim.7z.

#### 3.1.3 Statistical testing

For single-case data analyzed in simulations, custom statistical testing was introduced. Connectivity results were statistically tested using bootstrap statistics against the baseline (zero connectivity) threshold. First, the baseline had to be determined to treat all values below this value as nonsignificant. The procedure using a surrogate data approach was as follows:

- The surrogate data was prepared by randomly mixing signal samples in each data channel separately. This way, all dependencies between signals were canceled while preserving the original distribution of signals’ samples and their variances.
- The DTF functions were calculated for the surrogate data. The single maximal value of the DTFs over all signal combinations and all frequency points was determined. This way, a conservative measure for the analyzed signals has been provided, with only one statistical test performed.
- The above two steps were repeated 1000 times, producing 1000 values of maximal DTF values obtained for signals with no relations between them.
- The threshold was chosen at the 99th percentile of the distribution of the maximal DTF values from the previous steps.

The values of the connectivity of the analyzed signals were tested using a bootstrap method. The procedure used virtual segmenting to treat them as multi-trial data.

- From the original set of experimental trials, another set of the same size was drawn, with replacement.
- DTF values were estimated for the new set of trials.
- The above two steps were repeated 1000 times, each time for a different sampled set of trials.
- From the resulting distribution of DTF values from all samplings, we produced corridors of confidence symmetrically cutting the distributions from the previous step at a chosen significance level (99%), namely at the 0.5th and 99.5th percentiles.

Finally, the connectivity values were considered significant only when the confidence intervals were entirely located above the threshold value determined in the first step.

#### 3.1.4 Results

**FIGURE 4:**
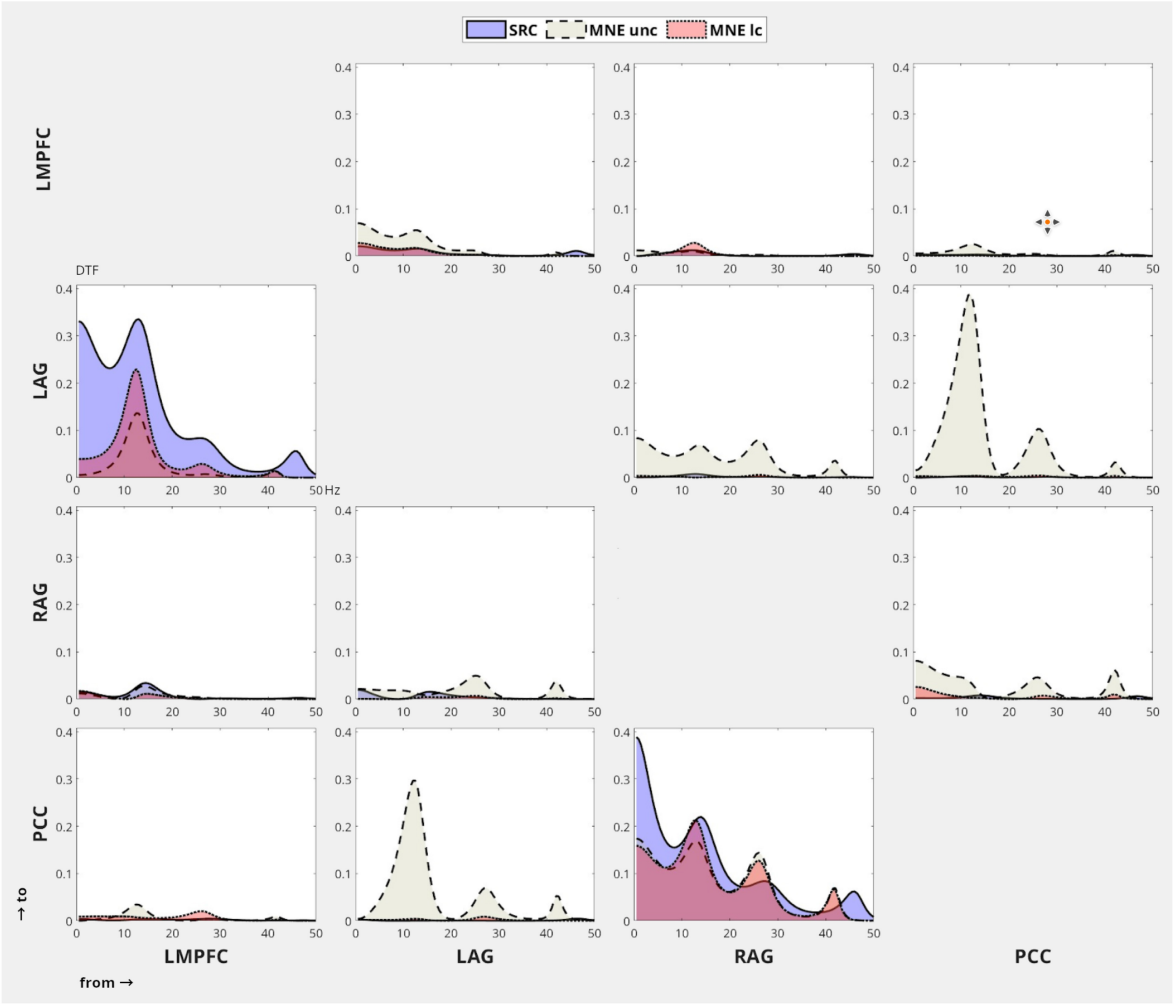
Simulation results. The estimated NDTF connectivity is shown for: (i) prepared signals that were used in forward calculations to simulate MEG signals (SRC); (ii) simulated MEG signals (MNE unc); (iii) simulated EEG signals after the leakage correction (MNE lc). The direction of connections should be read from x (columns) to y (rows).

Figure 4 shows the comparisons between DTF values obtained from the original source signals (SRC; marked in violet) and after the forward modeling and ASCT analysis for uncorrected (MNE-unc; yellow) and leakage-corrected signals (MNE-lc; red). In the case of source signals, two connections, where the interdependence was injected, namely LMPFC→LAG and RAG→PCC, are indeed visible as the elevated values of directed connectivity that cover the whole analyzed frequency window. At the same time, other directions hardly show any values deviating from zero, which confirms that DTF analysis correctly recognized experimental manipulation. In contrast, the reconstructed signals that did not undergo leakage correction show a pattern of connectivity that strongly deviates from those of source signals. Most strikingly, high DTF values were revealed in several directions where no dependence actually existed. Some of them are characterized by peaks even higher than the maximum amplitudes of LMPFC→LAG and RAG→PCC directions. Importantly, after leakage correction, the connectivity pattern again resembles the injected dependence of source signals, with some differences in the overall amplitude of DTF, especially for low frequencies for the LMPFC→LAG direction.

**FIGURE 5:**
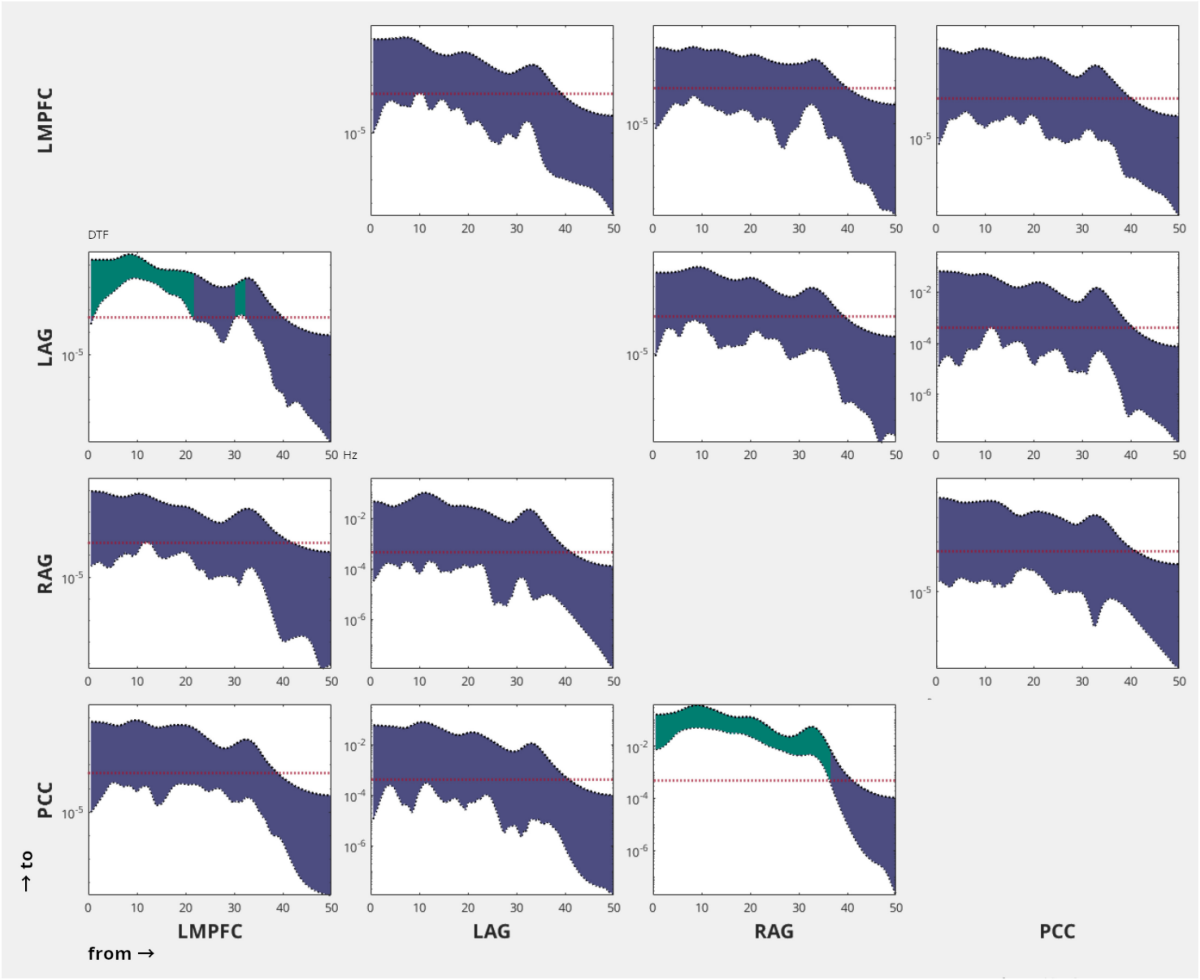
Statistics of the connectivity analysis of the simulated MEG signal with leakage correction. Plots show the lower (1st) and upper (99th) percentiles of bootstrap statistics against the baseline (zero) level determined by data shuffling (presented as a red dotted line). Significant connectivity (the whole CI is above the baseline level) is marked with green color. A log scale was used to better visualize near-zero baseline values. The direction of connections should be read from x (columns) to y (rows).

As the DTF estimation of uncorrected signals was apparently incorrect, only the leakage-corrected version was further analyzed in statistical tests. These results are presented in Figure 5. Plots show lower (0.5th) and upper (99.5th) percentiles of bootstrap statistics against the baseline (zero) level determined by data shuffling. Significant connectivity, i.e., the frequency range where the whole connectivity confidence interval was above the baseline level, is marked in green. Only two connections, LMPFC→LAG and RAG→PCC, passed significance tests. The connectivity was significant in the theta, alpha, and low and middle beta for the LMPFC→LAG direction and in the theta, alpha, beta, and low gamma in the RAG→PCC direction. No other directions were significant, which exactly matches the original source signal dependence.

### 3.2 Realistic evoked EEG data: the Eriksen flanker task

The second test was based on the real EEG recording during the Eriksen flanker task (Eriksen, 1995). This task is well-established in cognitive neuroscience for eliciting conflict-related effects, providing a robust framework for testing proposed EEG effective connectivity measures. In this task, participants respond to a central target stimulus (e.g., an arrow) flanked by peripheral stimuli (e.g., additional arrows) that can either point in the same direction (congruent trials) or the opposite direction (incongruent trials). Conflict arises on incongruent trials due to the competing information between the target and flanker stimuli, requiring participants to suppress the interfering flankers and focus on the target. This interference effect reliably engages cognitive control networks, making it ideal for studying brain connectivity during conflict processing.

Neurophysiologically, ongoing conflict engages a distributed network of cortical regions, where the detailed patterns of communication are only partially revealed. The anterior cingulate cortex (ACC), being a part of the salience network (SN), detects conflict and signals the need for increased cognitive control. This process also involves the anterior insula (aIns), a potential core hub of the SN (Cai et al., 2016). The information on the need for executive control enhancement is sent to the dorsolateral prefrontal cortex (dlPFC), which further implements the top-down control over the number of cortical regions (MacDonald et al., 2000; Botvinick et al., 2004). At the same time, the ACC is thought to receive input from the dlPFC and other regions, integrating contextual and task-related information to refine conflict detection and adjust control demands. Eventually, enhanced top-down connectivity within the fronto-parietal network–including the dlPFC signaling to the posterior parietal cortex (PPC)–is crucial for the successful implementation of goal-directed behaviors (Dosenbach et al., 2007; Ligeza et al., 2016).

Considering a strong involvement of the two above-mentioned large-scale brain networks in conflict resolution, the Flanker task can serve as a tool to verify the sensitivity of the ASCT analysis to cognitive control elicitation. The conflict-related increase in connectivity is expected after incongruent trials, reflecting the increased demand for attentional control. The growth of outflow from the anterior insula and cingulate cortex is expected, targeting the dorsolateral cortex and other considered regions. At the same time, increased top-down from the dorsolateral cortex is expected toward the parietal cortex, functionally linked to attentional control. The communication within the SN is primarily expected in the theta range, often considered a neural marker of conflict monitoring, while the frontoparietal network (FPN) is predicted to show effects in a wider range, in frequencies from theta to beta. However, the existing data tends to be inconsistent in this latter regard (Cavanagh and Frank, 2014; Ligeza et al., 2016, 2024; Kam et al., 2019; Spadone et al., 2021b; Wyczesany et al., 2025a),

#### 3.2.1 Data acquisition and analysis

The procedure has been approved by the Ethical Committee of the Institute of Psychology, Jagiellonian University (KE/59_2022; 10.11.2022). The privacy rights of the participant have been observed, while informed consent was obtained before the procedure. Data from a single participant were recorded using the 64-channel Biosemi Active Two device during the modified flanker task, as in Ligeza et al. (2018). Four additional electrodes were used as EOG leads and two more for mastoid reference. The recording was sampled at 1024 Hz and filtered with the windowed sinc finite impulse response (FIR) filters: high-pass (freq 2 Hz, order 3380) and low-pass (freq 47 Hz, order 1128) and downsampled to 256 Hz. Epochs were defined as 1-s segments relative to the target onset. Artifact rejection was performed using the following settings: channel and trial variance threshold = 7, range threshold = 250 μV, and muscle z-threshold = 22. This resulted in 4 trials removed due to muscle artifacts out of 275. ICA1 artifact rejection was performed with the following settings: ICA steps = 150, sensor map *z*-value = 7, single IC variance threshold = 20, multiple (6) ICs threshold = 7. These parameters were already empirically determined; see the section “4 User manual and guidelines.” At this stage, the P7 sensor and one more trial were removed. Then, the fastICA algorithm was repeated 20 times with tanh non-linearity and the number of steps set to 250. After the classification of the resulting components, the optimal ICA realization was selected. Resulting brain ICs were localized using the SimBio 5-layer head model and source model based on the SPM8 template brain as available in FieldTrip using the MNE method with depth weighting d set to 0.5. Lambda regularization was estimated based on the noise level equal to 1.5, as previously determined as optimal for this measurement environment. The following ROIs were selected as in Cai et al. (2016): the dorsal anterior cingulate (dACC: MNI: 7, 18, 33); the right anterior insula (raIns; MNI: 37, 16, −2); the right dorsolateral cortex (rDL; MNI: 58, 18, 44); the right ventrolateral cortex (rVL; MNI: 42, 25, 14); the right posterior parietal cortex (rPPC; MNI: 48, −52, 50). Reconstructed ROI signals were reduced by retaining the first PCA component, and the leakage correction was applied. DTF estimation was computed on the 0–0.5 s epochs relative to target onset separately for congruent (CONG) and incongruent (INCO) trials in the theta to gamma frequency range with the MVAR model order set to 7. Statistical tests were performed according to the procedure described in the previous simulation using the 0.001 p-level. The raw and processed data are available at https://atlantis.psychologia.uj.edu.pl/data/Flanker.7z.

To compare the results obtained with prior localization of ICs and the classic approach, where localization was computed over the complete sensor signals, an additional analysis was performed with similar settings. It included the same preprocessing and artifact rejection as in the original analysis, so the channel and trial rejection included exactly the same portions of data. Also, ICs originally classified as bad were subtracted from the signal. The main difference was that MNE was applied to the sensor signals, which directly yielded the requested ROI signals. Further analysis included PCA, leakage correction, and connectivity estimation, performed in the same manner as in the main analysis.

#### 3.2.2 Results

**FIGURE 6:**
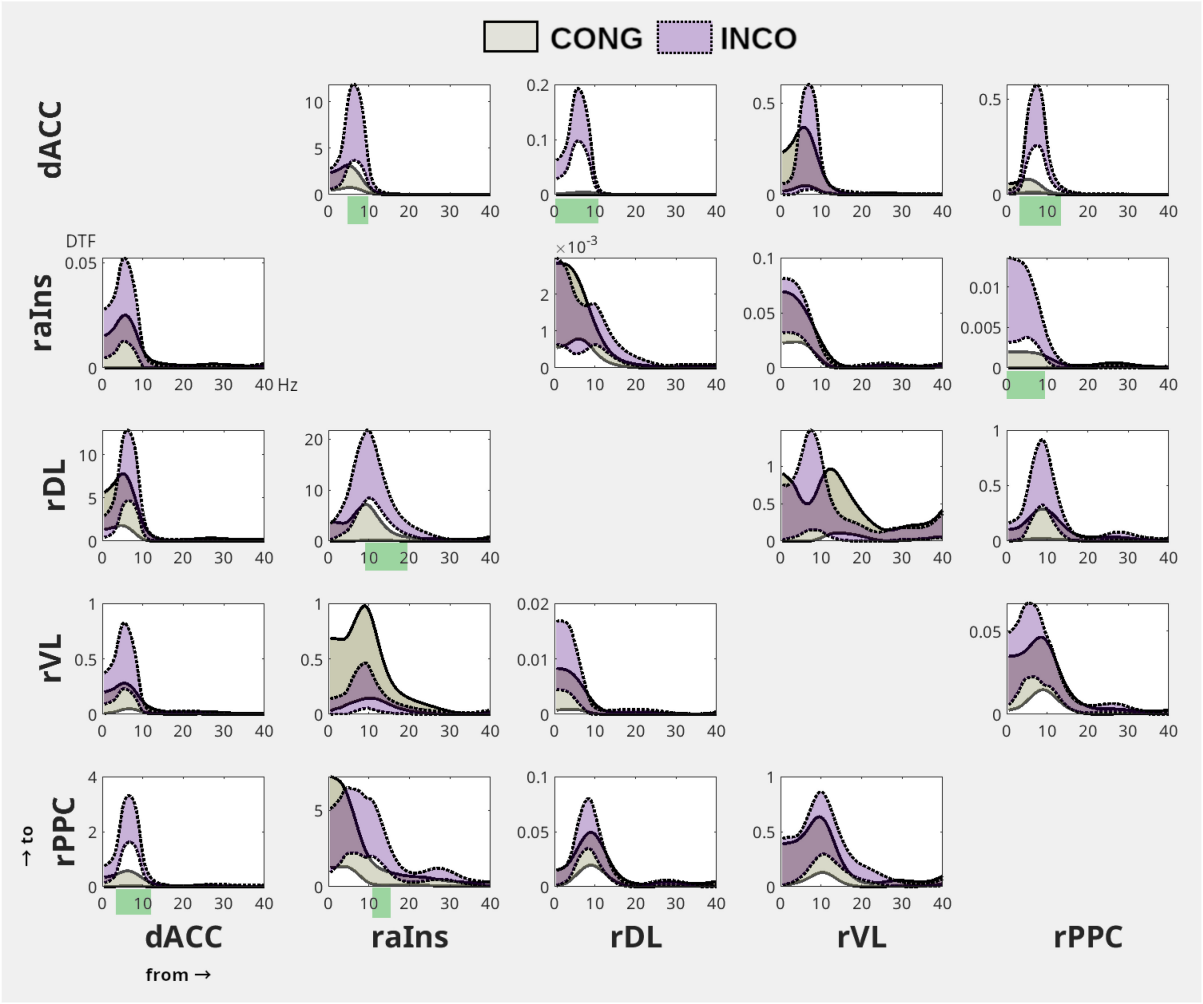
Statistics of the connectivity analysis of the single subject Flanker task EEG data. Plots show leakage-corrected DTF values for the lower (1st) and upper (99th) percentiles of bootstrap statistics for both congruent (brown) and incongruent (violet) trials. Green areas mark frequency ranges of significance. The direction of connections should be read from x (columns) to y (rows).

**FIGURE 7:**
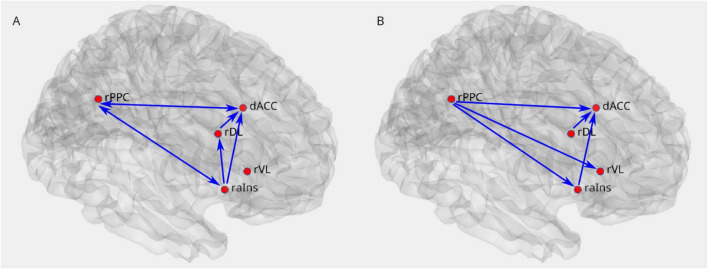
Connectivity plots for Flanker data analysis with separate localization of independent components **(A)** and localization applied over the complex signal **(B)**.

The comparisons of connectivity between INCO and CONG trials demonstrated an overall increase of connectivity in the whole analyzed network, mostly visible in the lower frequency range, from theta to alpha, with some directions also showing beta frequency changes. Statistical tests revealed significance for the following directions: dACC→rPPC in the theta frequency range, raIns→rPPC in the lower beta range, raIns→rDL in the lower beta range, raIns→dACC in the high theta and low alpha range, rDL→dACC in the theta and alpha range, rPCC→raIns in the theta range, and rPCC→dACC in the theta to alpha range. There were no significant differences showing an increase for CONG compared to INCO trials. The plots of DTF values as a function of frequency with statistical confidence intervals are presented in Figures 6, 7A.

**FIGURE 8:**
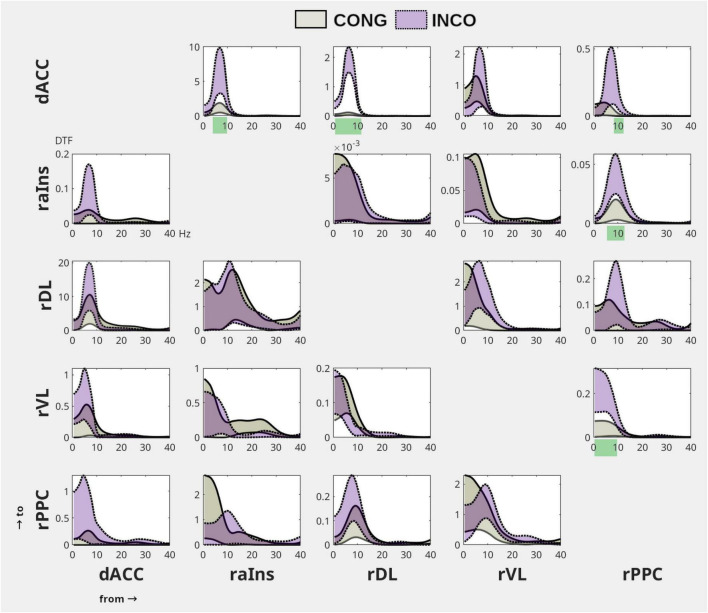
Statistics of the connectivity analysis where MNE was applied over the complex electrode signals (without prior ICA decomposition). Plots show leakage-corrected DTF values for the lower (1st) and upper (99th) percentiles of bootstrap statistics for both congruent (brown) and incongruent (violet) trials. Green areas mark frequency ranges of significance. The direction of connections should be read from x (columns) to y (rows).

The results of the analysis of conflict-related effects, where the localization was performed on the complex sensor signal instead of IC topographies, are depicted in Figures 7B, 8. As can be seen, many expected effects noted previously were not significant in this case.

### 3.3 Discussion

#### 3.3.1 Simulated MEG data

The relationships originally introduced in the simulation (LMPFC→LAG, RAG→PCC) are clearly seen in the connectivity of original source signals. At the same time, estimated DTF values remain very low for other directions. This manipulation check confirmed the internal structure of pre-prepared signals used here. The analysis of simulated MEG data, performed using the ASCT processing pipeline, revealed that the results with leakage-uncorrected signals, apart from true connections, showed some spurious effects that were non-existent in the simulated sources (especially LAG→PCC and PCC→LAG). Importantly, after the leakage correction, the connectivity results again correctly follow the introduced directionality, with no spurious connections present.

The results clearly indicate that the leakage correction is a critical step in the analysis and needs to be routinely applied prior to DTF application. Without it, zero lag correlations that are introduced during the source reconstruction as a spread of their spatial distribution seriously compromise the estimation of connectivity. Overall, presented results demonstrate that the proposed pipeline can accurately estimate connectivity patterns that were embedded in the signals.

#### 3.3.2 Eriksen flanker task EEG data

The comparison of directed connectivity between selected ROIs estimated for incongruent and congruent trials shows an apparent increase in overall DTF values. Several connections turned out to be significant at the relatively high *p*-level of 0.001, which suggests a notable effect of strengthening the connections when response control had to be intensified.

Outflows from the dACC show a relative increase visible in a low-frequency range, up to 10 Hz approx.; however, only the dACC→rPPC effect was found significant. This connection may play a role in modulating the sensory processing, as suggested by some data (Crottaz-Herbette and Menon, 2006). At the same time, the right anterior insula revealed three significant effects on outflows, targeting dACC, rDL, and rPPC. The raIns→dACC link can be considered intrinsic for the SN, primarily involved in the detection of events that require enhancement of cognitive control. The direction of the effect confirms a more recent view on the hierarchic structure of the SN, where the insula primarily signals to the cingulate cortex (Cai et al., 2016). Moreover, in line with the literature, this communication is indeed realized in low frequencies (Simmons et al., 2006; Clayton et al., 2015; Adamczyk and Wyczesany, 2023). Other connections from the anterior insula included these targeting the FPN (raIns→rDL and raIns→rPPC) which were found significant for higher frequencies, covering the high alpha and low beta range. More numerous outflow effects for raIns than for dACC can support a more recent view on the former as a core node that enforces causal influence on other brain areas when cognitive control is required (Menon and Uddin, 2010; Molnar-Szakacs and Uddin, 2022; Schimmelpfennig et al., 2023).

We could not confirm a significant conflict-evoked effect postulated as a top-down influence within the FPN, from the dorsolateral area to the posterior parietal cortex. However, a strong flow was apparent for the rDL→dACC direction for incongruent trials. This connection nearly drops to zero for the congruent condition. This observation can be interpreted as the causal influence of the FPN over the SN, related to signaling the rapid changes in cognitive control requirements (Dosenbach et al., 2007). It is especially interesting, as such a view on the FPN-SN interaction is still being disputed (Chen et al., 2016).

Interestingly, our pattern of outcomes closely resembles the results obtained by Cai et al. (2016), who analyzed the Flanker task data (together with other procedures that recruit enhanced cognitive control) with the fMRI Granger causal analysis. In both cases the raIns was recognized at the highest level of hierarchy among analyzed regions. The correspondence with the outcomes obtained using a different method supports the validity of the ASCT approach in analyzing evoked response paradigms.

Importantly, the additional analysis with localization performed on the complex sensor signals only partially replicated the results obtained with the ASCT ICA-based approach. In particular, we could not see a vast increase in raIns outflows in the incongruent condition. Facing the recent literature support for the major role of this structure in cognitive control, a failure to observe it in the final outcomes could indicate lower sensitivity of the approach where complex brain activity is considered for localization. At the same time, it also advocates for using source separation prior to source reconstruction.

#### 3.3.3 Final conclusions and limitations/future directions

Concluding the first simulation, the pipeline was able to reveal the original connectivity structure present in the analyzed signals. Importantly, the leakage correction appears to be critical for the analysis of source-based directed connectivity, and no results should be considered unless the correction is applied. Otherwise, final results may be plagued with false positive connections. This issue certainly requires special attention to determine the conditions of optimal leakage correction performance. Our previous studies can provide some anecdotal support for the ROI number, around 6–8, to be properly handled (Spadone et al., 2021b; Adamczyk and Wyczesany, 2023), but more studies are required to evaluate the impact of the ROI number on the multivariate leakage correction and the MVAR performance.

Moreover, our outcomes support the approach where sources are localized after their prior separation as a proper way of handling complex brain signals. This, however, still requires more detailed investigation. On one hand, the resulting simplification of MNE input potentially enables more accurate solutions. On another, however, it should be remembered that residual activity that remains after the ICA separation is not included in the further analysis. Another loss of data takes place during the reconstruction of ROI signals, where only the first PCA component is retained. Nevertheless, PCA is a method here that ensures the linearity of the final reconstruction, necessary for proper DTF estimation.

Our results support the reliability of the proposed ASCT pipeline. They also demonstrate that the proper data processing preserves causal relationships between the signals (Kaminski and Blinowska, 2014). The pipeline has been validated for the analysis of brain network dynamics using both resting state and evoked response data. To enable replicable analysis, in the next section we present recommendations for the directed connectivity analysis of EEG/MEG data, which can be considered as a starting point for further analyses.

## 4 User manual and guidelines

The guidelines below are intended to better describe the practical approach to analyzing data using the ASCT. They also provide important recommendations for parameter setup and the description of their impact on the analysis process. Following these guidelines will help in maintaining the reliability of connectivity analysis. For the full and up-to-date manual, please follow the link https://atlantis.psychologia.uj.edu.pl.

### 4.1 Introduction

The entire data analysis is defined within a single setup file (ExSetup.m) that is used to set all the parameters and eventually call the ASCT functions. Sample setup files are included in the/templates directory of the toolbox. In the following sections, we cover the most important settings. A short description of parameters is also included in the comments in the setup file.

The first section of settings controls the run of the analysis. It includes the main directory (rc.expFolder) with raw data subfolders (rc.dataRawFolder) and raw data format (rc.dataFormat). Importantly, rc.analysisName defines a separate analysis that will be saved in a subfolder of the same name. Within the main experimental folder, multiple analysis versions can be stored. This allows for multiverse analyses with alternate settings or studying different aspects of the same recording (for example, stimuli and response analyzed separately). This section also includes MRI parameters, which are optional and are described below. Parallel computing can be enabled with rc.useParallel (equal to the number of CPU cores to be used, “0” to disable), which boosts processing speed at the cost of increased memory demand.

By default, all datasets present in the relevant directories are processed. To limit this range (typically needed for testing purposes when setting up a new analysis), begId and endId can be provided (they represent the indexes of the files; to disable this feature, both need to be commented out). The current stages of data processing are selected with the rc.load and rc.stop parameters, which define the required starting and final analysis levels. The whole analysis is divided into the following steps (the name suffixes for resulting data files and target directories are provided relative to main analysis directory):

0: raw file read and preprocessing

   /1_PREPROC/…PREP.mat,…PREP_EOGcorr.mat

1: segmentation

   /2_SEGMENTED/…_SEGM.mat,…_SEGM_EOG

   corr.mat

2: sensor space artifact rejection

   /3_ARTIFREE/…_ARJ.mat

3: ICA1 decomposition

   /4_ICA1/…ICA1_decomp.mat

4: ICA1 artifact rejection

   /5_ICA1AR/…ICA1_arj.mat

5: ICA2 ‘clean’ decomposition

   /6_ICA2/…ica2.mat

6: IC classification

   /6_ICA2/…ica2.mat

7: realignment, head model, source model

   ([rc.mriRawFolder]/[dsname]/SC/…

   with multiple output files if MRI is

   used);

8: leadfield and localization

   /7_SOURCEACT/icloc/…icloc.mat

9: ROI signal reconstruction for all data configurations

   /7_SOURCEACT/DC../unc/…sigroi_unc.mat

   (uncorrected)

   /7_SOURCEACT/DC../unc/…sigroi_lc.mat

   (leakage-corrected)

10: connectivity estimation

   /8_CONN

   with multiple output files

11: statistics, visualization and data export

   /9_STAT 

   with multiple output files

### 4.2 Data import

Currently supported file types are: *EEGlab ‘set’*, *Biosemi ‘bdf’* (EEG), *CTF*, and *ITAB* (MEG). To properly recognize data structure, sensor indexes as in the raw files need to be provided: chan.meeg for actual EEG/MEG channels, chan.ref which are the reference electrodes for EEG or additional MEG sensors for noise balancing. If available, additional electric leads like EOG and ECG can be defined in the chan.elec parameter, which are beneficial for further artifact rejection and brain source classification. EOG channels need to be separately defined by the chan.eog parameter (apart from being included as electric leads). Electrode positions can be guessed based on name (for the 10–20 system), read from file headers, or provided as an external elc/mat text file in the chan.elcFile parameter.

### 4.3 Experimental design

ASCT recognizes subject id, group, and session codes directly from the file names. Therefore, the names need to be of the same length and include relevant codes on the positions defined by the design.idPosStart and design.idPosEnd parameters. Id has to be unique for a subject. Groups and sessions are coded in a similar way.

Experimental design definition includes: seg.studyType that differentiates between resting state (‘RS’) or event-related (’ER’) paradigms; seg.preStim and seg.postStim that mark the start and the end of the requested data epochs relative to target stimuli onsets. For the RS analysis, usually long data segments are virtually cut into smaller, non-overlapping epochs for the purpose of artifact rejection, as defined by the seg.rsSegm parameter (typically 2–5 s in length, to provide at least 30 segments, which will result in a pool big enough to estimate signal statistics during artifact rejection). We recommend keeping the de-mean epochs option enabled (seg.demean = 1) which will help to obtain IC signal without significant steps at epoch borders. This option overrides the baseline correction setting (seg.baseline).

Trigger code(s) and associated condition names have to be described in the design.COND(1) section, as in the following example, which defines two experimental conditions (‘congruent’ and ‘incongruent’), each marked by two different trigger codes:

design.COND(1).name = {‘congruent’, ‘incongruent’};

design.COND(1).trig = {{101, 102}, {201, 202} }.

The detailed configuration is partly dependent on the file format, as the storage of experimental events can differ. If additional recoding of triggers is needed before actual segmentation, a relevant script can be prepared to change the content of the trigger configuration trl and event structures that store the triggers’ type, code, and timing. Sample solutions can be found in the templates directory of the ASCT. To enable trigger recording, provide the name of the recoding function in the seg.recodeFn parameter.

Finally, data configurations (DC) are to be defined. DC can be explained as an elementary configuration of condition(s), group(s), and session(s) that will be further used as an element of statistical comparisons and data export. DCs do not need to be orthogonal. Please refer to the full user manual for examples.

### 4.4 Filtering

The windowed-sinc FIR filters are recommended for EEG/MEG signals (Widmann, 2012). As the connectivity analysis is estimated in a frequency range typically covering theta to beta frequencies, high-pass filtering is recommended to be set just below this range (1–2 Hz typically). Removing very low-frequency spectral components that dominate the EEG/MEG spectral power will help in ICA decomposition and in further MVAR model fitting. On the other hand, MVAR models benefit from some noise in the signal, and the low-pass filter cut-off should be set just below the line frequency (50 or 60 Hz). Filter order can be estimated with the formula: 3.3 × *f*_*s*_/*W*_*t*_ (*f*_*s*_ - sampling frequency, *W*_*t*_ - the width of the filter transition band). Summarizing, the recommended filter settings are: high-pass 2 Hz cutoff with a transition bandwidth of 0.5–1 Hz; low-pass 48 Hz cutoff with a 2–3 Hz transition bandwidth. All these parameters can be set in the filter section. Additionally, at this stage, the signal can be optionally downsampled to reduce resulting file sizes with the filter.resampleTo parameter (250–256 Hz is recommended).

### 4.5 Sensor space artifact rejection

In the first stage of artifact rejection (AR), bad channels are detected and removed. This step is especially important for EEG, where the average reference will be automatically applied to enable source reconstruction. Channel removal is based on the IQR thresholding of signal variance, which is set by the ar.chIQR parameter (recommended range 5–8). For EEG, if EOG data are available, eye movement correction is temporarily applied before calculation of channel variance to limit the EOG bias on statistical parameters of frontal derivations. ar.chRmLimit defines the upper acceptable limit for the number of removed channels. If exceeded, the whole dataset is removed (around one-fifth of the total sensor number is recommended here).

The next AR stage defines trial removal criteria. Detection is performed in three consecutive steps: excess trial variance (ar.varIQR, recommended value 5–8; a lower value can be set if EOG signals are available), difference between min/max amplitude (ar.rangeThr defines the actual threshold, around 150–300 μV for EEG), and muscle activity contamination (elevated spectral power for higher frequencies, typically > 35 Hz, defined by ar.muscThr as a *z*-score deviation; a value near or slightly above 20 is recommended).

The AR logs are stored in the 3_ARTIFREE subfolder, with detailed information on rejected channels and trials, together with their statistics. We recommend reviewing the accuracy of the actual settings, especially in a new measurement environment, by visual inspection of sample datasets. This can be obtained with the sc_plot function with segmented data as an argument. In this case, loading a segmented *SEGM.mat dataset from the 2_SEGMENTED subdirectory followed by the sc_plot(‘data_segm’) command will plot the data just before the rejection, and trial numbers in AR logs will refer to the trial indexes in this file. Verifying the channels/trials qualified for rejection and adjusting the settings accordingly will ensure optimal performance. Datasets after artifact rejection are saved in the 3_ARTIFREE directory as *ARJ.mat files.

### 4.6 Independent Component Analysis (ICA)

Independent Component Analysis settings include the decomposition mode (ica.approach): ‘deflation’ (that limits the number of resulting ICs) vs. the ‘symmetrical’ approach (where the number of ICs is equal to the number of channels). In the recommended deflation approach, its depth can be adjusted with ica.ICAx_maxSteps, which affects the number of resulting components. Higher values tell the ICA to more strongly seek out possible independent components in the portion of data not yet explained. This parameter can be empirically adjusted to obtain around 20–50 ICs, which typically requires 200–250 steps in the case of ICA2. It can be even lowered to 100–150 steps for ICA1, as the smaller number of ICs is enough for efficient artifact rejection at this stage. The ica.ICAx_nonlinearity allows for setting the nonlinear function; ‘tanh’ can perform slightly better and is recommended for ICA2, while ‘pow3’ is faster and is a good choice for ICA1.

To obtain an optimal separation, we recommend launching ICA multiple times (as defined by ica.ICAx_numIter). Three runs for ICA1 are usually sufficient for efficient AR, while ICA2, which is the actual decomposition used for further signal reconstruction, requires around 20–40 iterations. The selection of the best ICA decomposition is performed in step 6 (classification). Please note that ICA requires a sufficient amount of data for optimal performance (for details, see: Onton et al., 2006).

#### 4.6.1 ICA-based artifact rejection

Four parameters refer to the ICA1 AR. Noisy sensors usually produce ICs whose topography is strongly limited to this particular sensor. Such ICs can be detected with ar.ica_topoZThr which defines the z-threshold of sensor weights (a value of 7 is recommended). Trial removal is then performed: ar.ica_singleicZThr defines the threshold for IC signal variance deviation seen on a single IC (values between 15 and 20 are recommended), ar.ica_multiicZThr defines the threshold when the deviation is seen on multiple ICs at the same time (7–8 recommended), while ar.ica_multiicZCnt adjusts the number of these multiple ICs to look at (4–6 recommended). It is usually necessary to screen only the first components, so the ar.ica_ic2screen parameter is recommended to be set around 5–8 to limit the number of ICs to be checked.

### 4.7 Source localization

The settings for these stages of processing are grouped in the loc section. Here, the proper adjustment of the λ regularization parameter is crucial for MNE performance. Nevertheless, determining its optimal value can be practically challenging. When loc.norm is set to ‘lambda’, a fixed value has to be provided with the loc.lambda parameter, with a rough guess to start with around 0.1 (Leone et al., 2024). However, we recommend a dynamic estimation of λ based on IC parameters and the equivalent system noise. Since the IC maps are scaled (normalized) in the range [−100, 100], this equivalent system noise contains the contribution of errors on the channel calibration, and it is thus stable and linked to the specific recording system. In this case, loc.norm should be set to ‘cov’ and the proper noise level provided with loc.noiseLev. Empirical selection of this value is described in the section below. Additionally, to prevent the bias toward the superficial sources, which is characteristic of the classic MNE method, the depth weighting can be enabled with the loc.depthWeight parameter (recommended value 0.5).

#### 4.7.1 Empirical method for choosing an optimal lambda regularization parameter

It is recommended to optimize the regularization parameter λ by first determining the equivalent system noise level. With this approach, λ is adjusted based on IC parameters. Finding an optimal noise level value can be achieved by examining the quality of source localization performed on possibly clean sample data across different settings. This value can be considered specific for a given device and can be applied across different measurements with the same measurement environment.

**FIGURE 9:**
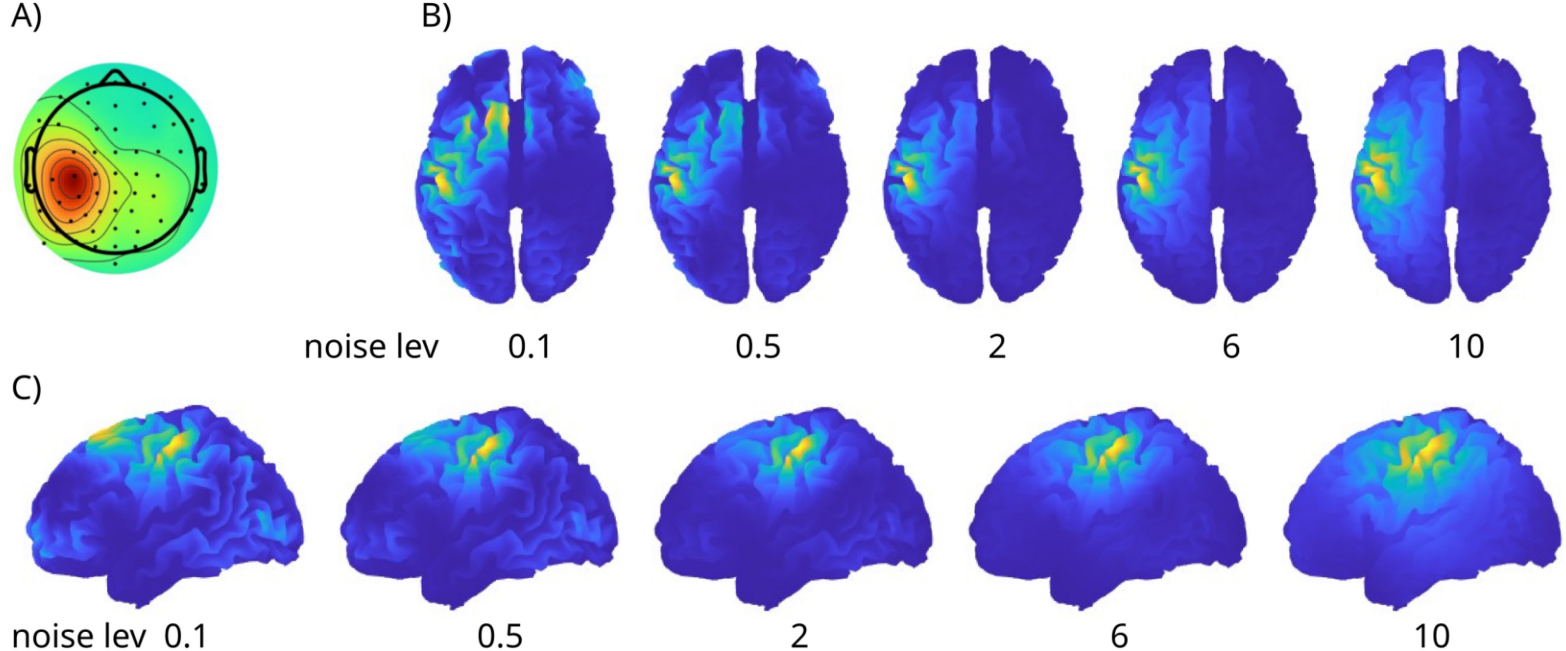
Optimization of the lambda parameter based on estimation of the system noise level. **(A)** Topography of a sample IC that was used for the optimization procedure; **(B,C)** top and right views of its source reconstruction performed with different values of the loc.noiseLev parameter. In this case, the system noise equal to 2 seems to be an optimal value; for lower values, the reconstruction of the component gets scattered, while higher values result in more spatial spread.

The prerequisite for this procedure is to select a dataset with some “good” ICs of brain origin that have consistent, smooth topographic images (to be checked in 5_ICA2/[dsname] as _BRAIN_BEST_ITER_x_MAPS.jpg file). It is recommended to select one of the subject’s first ICs due to their relatively high signal content. In ExSetup the following parameters have to be set: rc.lambdaScan = 1 (enables parameter scan mode), rc.load = 8, rc.stop = 8, loc.norm = ‘cov’. Moreover, beg_id and end_id should point at the selected dataset. After launching ExSetup in the scan mode, sc_lambda_est.m script is performed, where ICs to plot and scan range can be defined. It produces a set of source images saved in the 7_SOURCEACT/icloc/lambdafigs subdirectory. Examining the localization results, the optimal value of loc.noiseLev parameter can be determined. The optimal value would produce a possibly focal and unitary solution. Too high values will result in excess blur, i.e., spatially extended solutions, while too low values will make them fragmented and scattered in space. Sample scan results are depicted in Figure 9.

#### 4.7.2 Application of individual head- and source models

To improve the accuracy of source localization, individual head and source models are required to account for differences in head/brain shapes. Custom head models can be individually constructed from T1 MRI images. Relevant settings include T1 files location and format (rc.mriRawFolder, rc.mriExt, rc.mriCoordsys) and further loc.headmodelMethod. We recommend realistic 5-layer SimBio (Vorwerk et al., 2018) head models for EEG (loc.headmodelMethod = ‘simbio’) and a realistic homogenous head model for MEG (loc.headmodelMethod = ‘singleshell’). An additional shell script, tools/FS-recon.sh is provided for batch preparation of individual source models based on FreeSurfer cortical reconstruction (Fischl, 2012) and HCP-workbench downsampling of the cortical mesh (separate installation required), according to the procedure described in the relevant FieldTrip manual https://www.fieldtriptoolbox.org/tutorial/source/sourcemodel/.

If individual MRI images are not available, template (averaged) head and source models are used instead at the cost of decreased accuracy. In such a case, head and source models based on canonical SPM8 templates are used by default. Other template models can be used (for example, for children’s subjects), which need to be specified by the rc.headTemplate and rc.sourceTemplate parameters.

For EEG measurement, even more accuracy can be achieved with individual electrode positioning that can be obtained from 3D modeling devices. In order to use text files with individual electrode coordinates, the rc.elecRealPos parameter has to be specified to point at the directory with individual electrode mat files containing locations of sensors and fiducials in the same coordinate system saved in the FieldTrip format. For more detailed instruction on 3D electrode scanning, see: https://www.fieldtriptoolbox.org/tutorial/source/electrode/.

### 4.8 Signal reconstruction

The reconstruction of the brain signals is performed for the ROIs that are defined using a custom name (loc.ROI(x).name) and standardized MNI coordinates (loc.ROI(x).pos). Both the direct result of reconstruction and the one that underwent leakage correction are saved (corrected signals are stored separately for each DC in: 7_SOURCEACT/DCx/lc/…sigroi_lc.mat). Only the corrected signal should be used for further connectivity estimation, as the analysis of uncorrected signals is contaminated with spurious connectivity due to the spatial blurring of reconstructed sources. In the case of individual head and source models, they are warped to the MNI template before the signal is reconstructed. This provides a match with ROI definitions that have to be expressed in a standard MNI space. During the signal reconstruction, the con.decimate parameter enables downsampling the reconstructed signal by providing the factor for sample frequency division. It is intended to set the final sampling rate near the recommended value of 128 Hz (for example, if the sampling rate after preprocessing is 256 Hz, this parameter should be set to 2 at this point).

### 4.9 Connectivity estimation

Multivariate connectivity estimation is performed on reconstructed and leakage-corrected signals. The result of connectivity estimation is saved in matrices, whose three dimensions mark destination ROI, origin ROI, and frequency bins (in 0.5 Hz resolution). It is possible to create multiple versions of connectivity with different settings, which are stored in the 8_CONN/[con.conName] subdirectory. MVAR settings include model order (con.mvarOrd), which determines the number of samples that account for the prediction of model coefficients. The model order value is dependent on the actual sampling rate. For the recommended value around 128 Hz, we suggest fixing the model order *p* to the value around 7. This recommendation is mainly based on the observation that in a typical EEG/MEG signal we expect 2–4 frequency components (rhythms) to be present, and the frequency domain transfer matrix of an AR model allows us to describe *m* = p/2 frequency maxima in the single-channel spectrum. The long-term practice shows that for real EEG/MEG signals, popular criteria (AIC, FPE) are not always straightforward to use. The estimations they provide can vary between methods and suggest an optimal model order in the range of 4–15 (at the sampling frequency around 100–128 Hz) depending on the presence of specific rhythms. Also, they can be inconclusive, showing no local minimum in the function of *p*.

Before the DTF estimation, it is recommended to normalize ROI signals using their variance, which helps to alleviate a possible imbalance in signal strengths (con.varNorm = 1). By default, connectivity is estimated using all defined ROIs and using the full epoch as defined by seg settings. To limit these, con.chanSel can be used to define the ROI set to analyze (for all, set it to ‘1-’). When con.useRange is set to 1, only a fraction of the epoch defined will be taken into account during the connectivity estimation, as provided by con.timerangeStart and con.timerangeEnd parameters expressed in sec relative to stimuls onset.

### 4.10 Statistics, visualization, and data export

The simple statistics module is limited to pairwise significance tests between any defined data configurations. These configurations can include any combination of selected conditions, groups, or sessions. The effects of these contrasts are visualized in matrices of *n_*r*_ x n_*r*_* size, showing thresholded *p*-levels for all directional connections between defined ROIs. Boxplots presenting connectivity distribution are also saved for significant directions. Results are also rendered on 3D brain images, which can be customized and rotated.

To perform statistical contrasts and export connectivity data, a pair of DC (data configurations) needs to be defined by the con.contrast2test parameter. The frequency range in Hz for which connectivity is to be estimated is defined with the con.freqRange and the associated name (visible in the export files) by the con.subTitle variable. The significance threshold (used for visualization purposes) can be applied with the con.pThresh. To choose ‘within’ or ‘between’ statistical comparisons, con.testType needs to be set, while one- or two-tailed tests are defined by the con.oneTailed option. As connectivity data tends to have skewed distribution, it is recommended to set con.isParametric to 0, which will result in performing the Wilcoxon signed rank test. To remove outlier values that can be present in connectivity data, the IQR rejection methods can be applied before statistical testing by setting the con.iqr (recommended value around 3). Some parameters can include multiple values. Then, they are sequentially read, and the multiple calculations are performed within a single statistical run (con.contrast2test, con.freqRange and con.subTitle, con.pThresh, con.iqr). Export files include spreadsheets with connectivity data, significance matrices, and boxplots for directions that passed the significance test. They can be used for advanced statistical modeling using external software.

## Data Availability

The datasets presented in this study can be found in online repositories. The names of the repository/repositories and accession number(s) can be found below: https://atlantis.psychologia.uj.edu.pl/data/Flanker.7z, http://atlantis.psychologia.uj.edu.pl/data/Sim.7z.

## Appendix

### The origin of EEG signal

The electric field propagates through the extracellular medium (tissues) according to the fundamental Maxwell equations, the equation of continuity of current, ∇●*j* = 0 and Ohm’s law *j*_*v*_ = σ*E*. These laws lead to a propagation equation of electric potential *V*

$$S\,\left(r\right)=\nabla ●j_{v}=-\nabla ●\left(\sigma \,\nabla V\right)$$
(12)

where *S*(r) is a current source density, understood as a measure of inflow or outflow of volume currents.

### The origin of MEG signal

Magnetic field is generated by the currents according to Biot-Savart law

$$B=\frac{\mu \,0}{4\,\pi }\,\int \frac{j\,\left(r\right)\times R}{R^{3}}\,dv$$
(13)

Under the assumption of sufficiently fast decay of current density with distance, the last equation can be written as:

$$B\,\left(r\right)=\frac{\mu \,0}{4\,\pi }\,\int \left[j_{p}\,\left(r\right)+V\,\nabla \sigma \,\left(r\right)\right]\times \frac{R}{R^{3}}\,dv$$
(14)

For a homogeneous medium, the second term in the equation vanishes; therefore, only primary currents contribute to the magnetic field.

### Depth weighting

The depth weighting is obtained by the modification of the MNE cost function.

The cost function (see Equation 3) now takes the form:

$$F\,\left(x\right)={\left|\mathrm{Lx}-y\right|}^{2}+\lambda \,{\left|\mathrm{Wx}\right|}^{2}$$
(15)

where W is a depth-weighting matrix, which introduces penalties for sources with strong leadfield elements (which is the case for superficial sources). In practice, W includes diagonal elements that are proportional to the strength of a particular source. i.e., an L^2^ norm of the corresponding column of the lead field matrix.

The solution of the optimization problem takes the form:

$$\hat{x}={\left(W^{\mathrm{TW}}\right)}^{-1}\,L^{T}\,{\left(L\,{\left(W^{\mathrm{TW}}\right)}^{-1}\,L^{T}+\lambda \,I\right)}^{-1}\,y$$
(16)

where (W^TW^)^−1^ = R is sometimes called an *a priori* source covariance matrix.
